# Evidence and evidence gaps in the treatment of Eustachian tube dysfunction and otitis media

**DOI:** 10.3205/cto000132

**Published:** 2016-12-15

**Authors:** Magnus Teschner

**Affiliations:** 1 Department of Otolaryngology, Head and Neck Surgery, Hannover Medical School, Hannover, Germany

**Keywords:** otitis media, Eustachian tube dysfunction, evidence, evidence gaps, antibiotic therapy

## Abstract

Evidence-based medicine is an approach to medical treatment intended to optimize patient-oriented decision-making on the basis of empirically proven effectiveness. For this purpose, a classification system has been established to categorize studies – and hence therapy options – in respect of associated evidence according to defined criteria.

The Eustachian tube connects the nasopharynx with the middle ear cavity. Its key function is to ensure middle ear ventilation. Compromised ventilation results in inflammatory middle ear disorders. Numerous evidence-based therapy options are available for the treatment of impaired middle ear ventilation and otitis media, the main therapeutic approach being antibiotic treatment. More recent procedures such as balloon dilation of the Eustachian tube have also shown initial success but must undergo further evaluation with regard to evidence. There is, as yet, no evidence for some of the other long-established procedures.

Owing to the multitude of variables, the classification of evidence levels for various treatment approaches calls for highly diversified assessment. Numerous evidence-based studies are therefore necessary in order to evaluate the evidence pertaining to existing and future therapy solutions for impaired middle ear ventilation and otitis media. If this need is addressed, a wealth of implications can be expected for therapeutic approaches in the years to come.

## 1 Introduction

### 1.1 Evidence in otolaryngology

The idea of evidence-based medicine can already be found in the 18^th^ century when British physicians developed the concept of “medical arithmetic” requiring abandonment of superstition and renunciation of useless drugs in the conception of therapeutic approaches. Instead, an empirically elaborated evidence should be applied which is generated by means of scientific studies and systematic observation [12]. According to recent knowledge, the term of “evidence in medicine” was first used in 1793 by the Scottish physician George Fordyce [123].

The current evidence-based medicine is based among others on the work of David Sackett who was lecturer at the Department of Clinical Epidemiology and Biostatistics of the McMaster University, Hamilton, Canada, as founding director. Furthermore, it is based on the work of the American physician and mathematician Alvan R. Feinstein with his publication entitled “Clinical Judgement” that appeared in 1967 [39] and on the publication of the British epidemiologist Archie Cochrane entitled “Effectiveness and efficiency: random reflections on health services” [25]. Those publications led to an increasing acceptance of clinical epidemiology [6].

Today, evidence-based medicine means that decisions of medical treatment are made patient-oriented, possibly based on empirically proven effectiveness [133]. Hereby, the currently best evidence for or against decisions in the treatment of the individual patients should be applied. The basis is the most recent status of clinical medicine and published clinical trials that confirm or disprove an option [103].

The classification of evidence into evidence levels is an important aspect of evidence-based medicine. This classification is made based on a gradient: the higher the classification of evidence, the better the scientific justification for a resulting treatment recommendation. Thus the evidence level provides an orientation of the effectiveness of the therapeutic option which, however, must be critically questioned by the treating physician. This does not mean that all trials or therapeutic approaches of level 1 have to be considered as irrevocable facts and that all trials or therapeutic approaches of level 4 have to be ignored [18].

For categorization of evidence, various classification systems and assessment scales are available. Beside the “Strength-of-Recommendation Taxonomy (SORT)” with the classifications of A to C [34], there are also the “Guidelines of the Agency for Healthcare Research and Quality” (classifications of A, B, C, X), the “Guidelines of the US Preventive Services Task Force“ (classifications of A, B, C, D, E), the “Guidelines of the University of Michigan” (Practice Guideline with classification into A, B, C, D) [8], or the detailed “Classification Schemes of the Centre of Evidence-Based Medicine” of the University of Oxford [126]. Mostly acknowledged is the GRADE classification of the Grade Working Group [5], [38], [43], [45] (Table 1 (Tab. 1)). This latter classification will be used in this paper.

This paper will give an overview of evidence and evidence gaps regarding the treatment of impaired middle ear ventilation and otitis media. In this context, the focus will be limited on the clinic of acute otitis media and secretory otitis media. The aspects of chronic otitis media with defect of the tympanic membrane are included in the pathophysiological context. All therapeutic aspects of chronic otitis media are considered only marginally with regard to their evidence.

### 1.2 Anatomical basics

#### 1.2.1 Anatomical basics of the Eustachian tube

**Figure 1:**
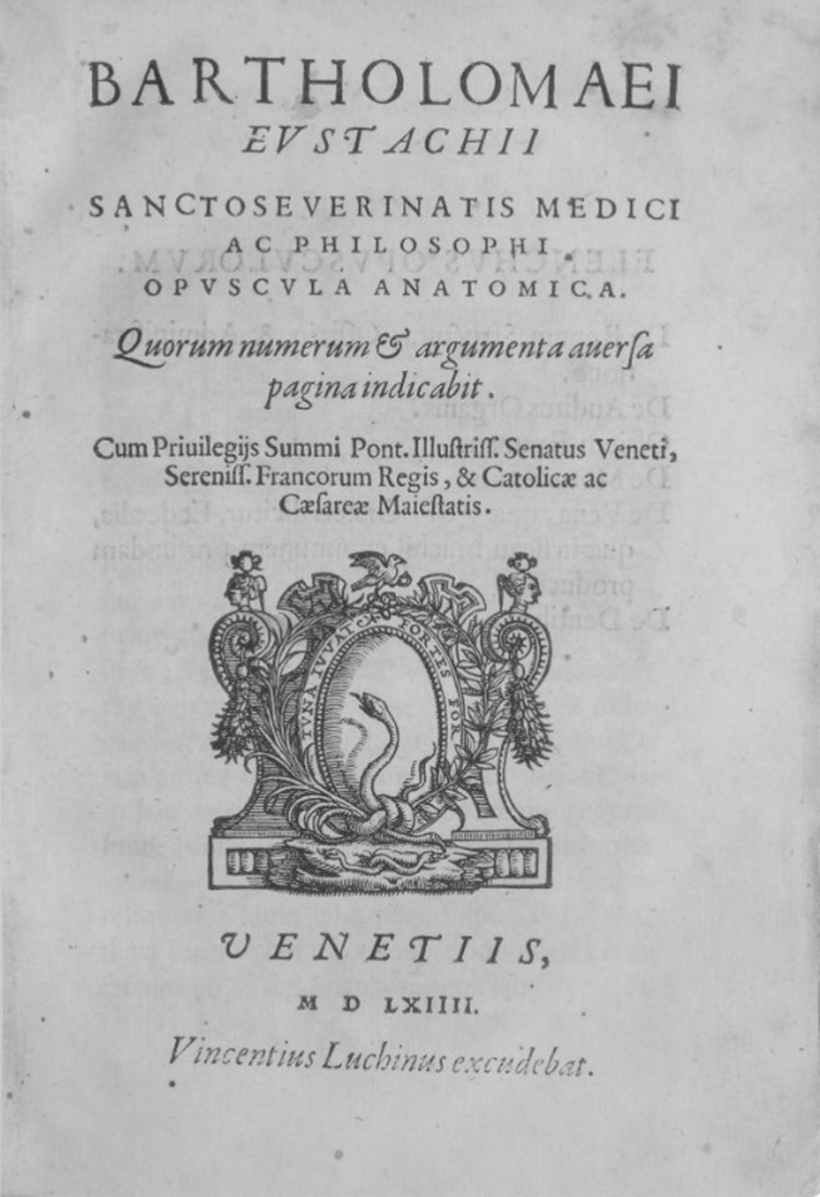
Opuscula anatomica by Bartolomeo Eustachi (source: B. Opuscula anatomica, Vincentius Luchinus. De auditus organis. October 1562, [37])

**Figure 2:**
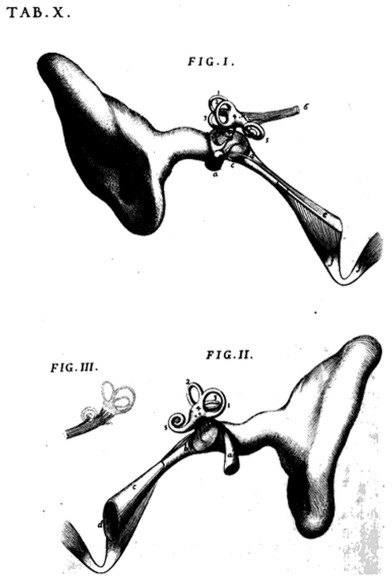
Drawing of the Eustachian tube by Antonio Maria Valsalva from 1717 (source: Valsalva AM. De Aura Humana Tractus. Trajecti ad Rhenum: apud Guillielmum vande Water, 1717, page 166, [127])

The term of “Eustachian tube” dates back to the Italian physician Bartolomeo Eustachi [37]. In 1562, he described in his multipart work entitled *Opuscula anatomica* the anatomical structure that is known today as Eustachian tube (“*De auditus organis*”) (Figure 1 (Fig. 1)). Also the Italian anatomist and physician Antonio Maria Valsalva focused on this anatomical connection between the nasopharynx and the middle ear and in the 18^th^ century he continued Eustachi’s explanations with further extensive descriptions of the anatomy as well as anatomical drawings of the Eustachian tube [127] (Figure 2 (Fig. 2)).

**Figure 3:**
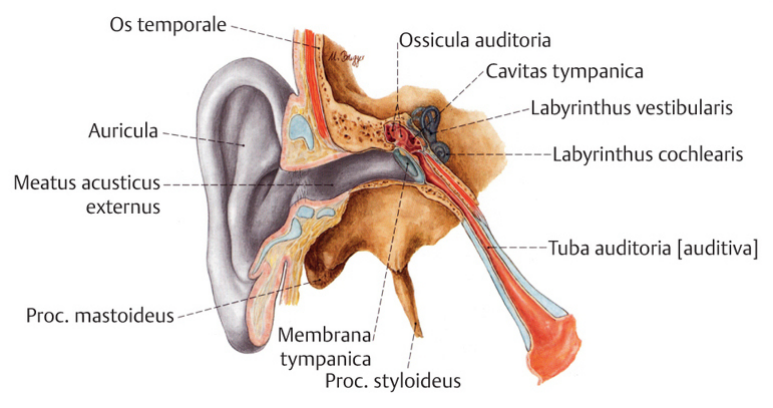
Graphical presentation of the anatomy of the Eustachian tube and the ear. Taken from: Paulsen, Waschke. Sobotta, Atlas der Anatomie des Menschen. 23th Edition, 2010 © Elsevier GmbH, Urban & Fischer, München [89]. Courtesy of the editor.

**Figure 4:**
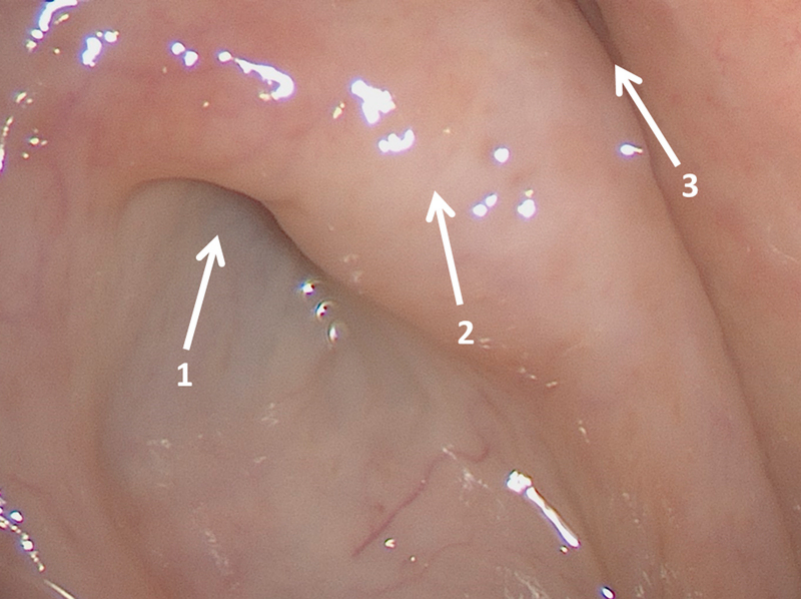
Transnasal endoscopic view (70° optic) into the right-sided nasopharynx. In the nasopharynx the pharyngeal ostium of the Eustachian tube is found (arrow 1). Posterior-superior is the torus tubarius (arrow 2) that limits the ostium. In dorsal direction, the fossa of Rosenmüller is found (arrow 3).

The Eustachian tube connects the nasopharynx with the middle ear. It consists of a cartilaginous and a bony part (Figure 3 (Fig. 3)). The cartilaginous portion is located proximal to the nasopharynx and is formed by a cartilage ring, which can be described as hook-shaped structure showing a high anatomical variability [85]. Connective tissue reinforces the Eustachian tube at the lateral and inferior parts. The torus tubarius forms the cartilaginous opening of the Eustachian tube to the nasopharynx (Figure 4 (Fig. 4)).

**Figure 5:**
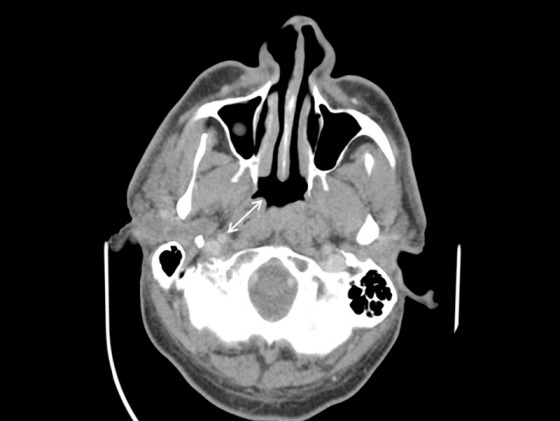
Computertomographic presentation of the distance of the torus tubarius to the internal carotid artery in axial reconstruction. The distance between the torus and the internal carotid artery amounts to an average of 23.5 mm (double arrow).

**Figure 6:**
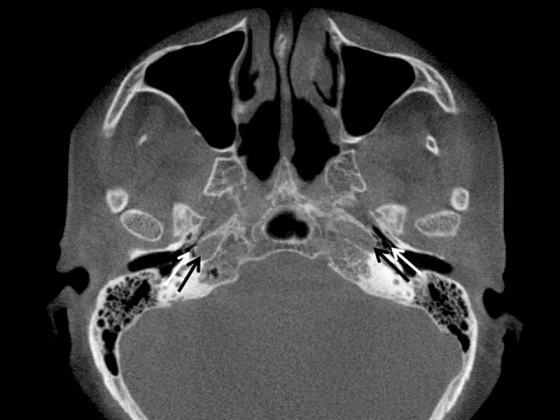
Computertomographic presentation of the distance between the bony tube canal and the internal carotid artery in axial reconstruction. The white arrows mark the lumen of the Eustachian tube, the black arrows mark the bony canal of the internal carotid artery.

The distance between the torus of the opening of the Eustachian tube and the bony canal of the internal carotid artery varies enormously and measures on average 23.5 mm (minimum 10.4 mm) (Figure 5 (Fig. 5)). Statistically significant anatomical differences with regard to gender are not known [10]. In its further course, the Eustachian tube is parallel to the internal carotid artery with bony borders (Figure 6 (Fig. 6)). In the area of the transition of the bony to the cartilaginous portion, the lumen of the Eustachian tube is narrowest [105]. The Eustachian tube is lined by respiratory epithelium. The flapping of the kinocilia is directed to the pharynx [105]. The part of the Eustachian tube that is suspected to be mostly responsible for a ventilation disorder is the part located near the tympanum [120].

#### 1.2.2 Anatomical basics of the middle ear

**Figure 7:**
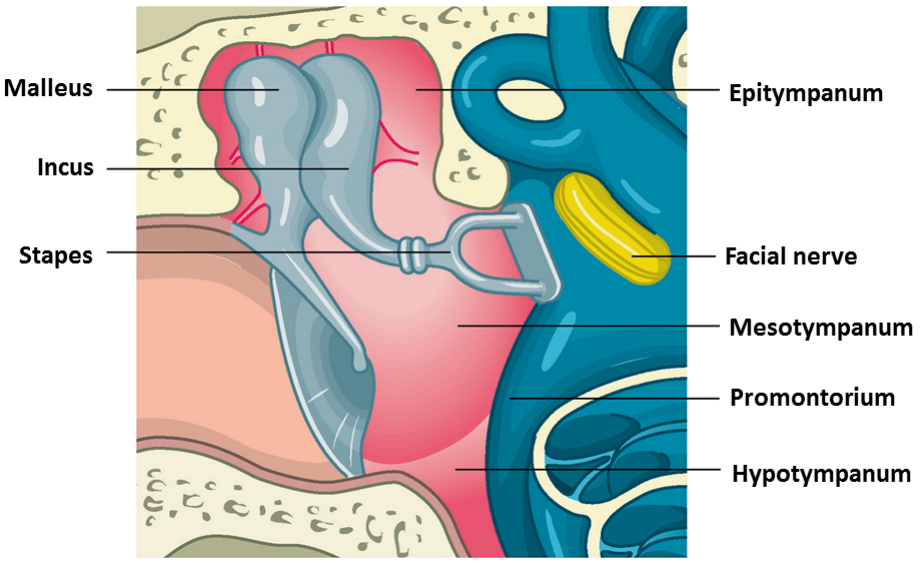
Scheme of the anatomy of the middle ear. It shows the tympanic membrane, the ossicular chain, the facial nerve, and parts of the cochlea and the semicircular canals (source and copyright: Lenarz T, Boeninghaus HG. HNO. 14th Edition, 2012, page 15, [64]. Courtesy of Springer).

The middle ear consists of a bony cavity where the ossicular chain of malleus, incus, and stapes is found (Figure 3 (Fig. 3)). This cavity is also called tympanic cavity (cavum tympani) and measures about 20 mm in height, 10 mm in length, and is around 2 mm wide at its narrowest point between the promontorium and umbo (Figure 7 (Fig. 7)). The mucosa consists of a single-layer, flat to isoprismatic epithelium. In the neighborhood of the tube orifice, kinocilia are found. Below the epithelium, the highly vascularized lamina propria is seen [105]. The anatomical borders of the tympanic cavity consist of the paries tegmentalis, jugularis, labyrinthicus, membranaceus, mastoideus, and caroticus [115] (Table 2 (Tab. 2)).

### 1.3 Physiological basics

#### 1.3.1 Physiological basics of the Eustachian tube

The physiological function of the Eustachian tube is to secure pressure balance between environmental pressure and the pressure of the middle ear. This balancing is achieved by opening the Eustachian tube, which happens when the pressure gradient increases. Opening of the tube, however, can also be achieved by muscular activity, e.g. swallowing or yawning. The palatine tensor muscle and the palatine velar levator muscle start at the lamina membranacea. Activity widens the lumen of the Eustachian tube and negative pressure of the tympanic cavity can be balanced [105].

Furthermore, the medial pterygoid muscle is involved. Its role is to be a mobile hypomochlion of the palatine velar tensor muscle. Contraction of the medial pterygoid muscle increases the efficiency of the palatine velar tensor muscle, relaxation leads to a decrease [66], [67].

#### 1.3.2 Physiological basics of the middle ear

Via the ossicular chain the sound waves reaching the tympanic membrane are transferred to the perilymphatic space. Elastic ligaments allow swinging suspension. The centers of gravity and the pivot points of the ossicles meet in one point so that movements of the head do not cause relevant swinging and thus no noise perception occurs. The transmissions of low frequent and high frequent sound waves of the air are clearly poorer so that a high-pass filter (quasi-static movements) as well as a low-pass filter are observed. The ability of the eardrum to swing is depends largely on the pressure difference in comparison to the environment. Since the mucosa of the middle ear permanently absorbs air, there is a continuous negative pressure in the middle ear that is balanced by the opening of the Eustachian tube. If the pressure gradient increases or if there is no pressure compensation between the middle ear and the environmental pressure, the ability of sound conduction decreases [30].

The middle ear adapts the low acoustic impedance of the air to the high impedance of the cochlear liquid. The necessary pressure enhancement originates from the surface ratio of the ear drum to the footplate of the stapes of 17:1 as well as the leverage effect of the ossicles of 1.3. This leads to a pressure enhancement by factor 22. If this impedance adaption is missing, conductive hearing loss results [30], [113].

### 1.4 Pathophysiology of tube dysfunction and otitis media

The pathophysiological origins of tube dysfunction and otitis media are often bacterial infections developing on the floor of viral infections, adenoid hypertrophy, or even genetic predisposition.

#### 1.4.1 Microbiology of tube dysfunction and otitis media

The origin of bacterial inflammation of the middle ear may be a viral infection of the nasopharynx. This infection is often associated with tube dysfunction and can thus be considered as risk factor of acute otitis media. The viruses hereby induce an immune and inflammatory reaction of the Eustachian tube and the middle ear so that a bacterial superinfection leads to otitis media [23].

In nasopharyngeal swabs of patients with recurrent acute otitis media, significantly more rhinoviruses, bocaviruses, parainfluenza viruses, adenoviruses, and respiratory syncytial viruses were found compared to healthy children [133].

Furthermore, more than half of the otitis media cases revealed a positive virus test in the pharyngeal swabs. In a prospective longitudinal cohort study of 294 healthy children aged between 6 months and 3 years, 1,295 infection episodes of the upper respiratory tract (5.06 episodes per child per year) and 440 acute otitis media episodes (1.72 episodes per child per year) were observed during an interval of one year. For 864 infections of the upper respiratory tract, viral examinations were performed. In 63% a positive virus test was found. Most often, rhinoviruses and adenoviruses were observed. In 61% of the infections of the upper respiratory tract otitis media developed as complication. Categorized according to the pathogens, about half of the infections of the upper airways with adenoviruses, respiratory syncytial viruses, or corona viruses as well as one third of the infections with influenza viruses, parainfluenza viruses, enteroviruses, or rhinoviruses were accompanied by the complication of acute otitis media [24]. Also other trials found viruses in two third of all children with acute otitis media [81].

More recent investigations of the upper respiratory tract in children up to the age of one year showed that in about 25% of all viral infections of the upper respiratory tract acute otitis media occurs as complication. In cases of asymptomatic viral infection, however, no acute otitis media was observed. The incidence of acute otitis media was associated with an increasing age and infection with respiratory syncytial virus, rhinovirus, enterovirus, adenovirus, and bocavirus [22].

Investigations on bacterial co-colonization and viral interaction in the pathogenesis of acute otitis media revealed that the risk of acute otitis media was higher when patients had co-colonization with *Streptococcus pneumoniae* and *Moraxella catarrhalis* or *Haemophilus influenzae* and *Moraxella catarrhalis* in comparison to patients who had colonization only with *Streptococcus pneumoniae* or *Haemophilus influenzae* alone. Co-colonization with *Moraxella catarrhalis* seems to increase the risk of acute otitis media. Furthermore, an infection with respiratory syncytial virus may lead to acute otitis media even without bacterial infection of the nasopharynx [102]. 

Beside the respiratory syncytial virus and the influenza virus, also the human metapneumovirus is associated with an increased occurrence of acute otitis media [116].

The respiratory syncytial virus is the most frequently found virus in acute otitis media. Heikkinen et al. investigated the prevalence of different viruses in the tympanic secretion of children up to the age of 7 years with acute otitis media. In 41% the virus test was positive. The respiratory syncytial virus was most frequently occurring virus (74%), followed by parainfluenza virus (52%), and influenza virus (42%), enterovirus (11%) and adenovirus (4%) (with p≤0.01 for all comparisons) [48].

This viral infection is mostly followed by bacterial infection. Frequent bacterial pathogens of otitis media are *Haemophilus influenzae* or *Streptococcus pneumoniae*. Another pathogen that is often found is *Moraxella catarrhalis* [28], [121]. 

#### 1.4.2 Adenoid hypertrophy

The pharyngeal tonsil called adenoid belongs to the lymphatic ring (Waldeyer’s tonsillar ring). This lymphatic ring consists of mucosa-associated lymphatic tissue and as B cell organ it has an immunological function. Increase of the surface often leads to sagittal folding of the mucosa.

The hyperplasia of the pharyngeal tonsil develops from increasing reaction centers of the lymphatic tissue. The exact origin of this hyperplasia is not definitely clarified. It is suspected that a multifactorial development takes place while secretion stagnation occurs due to inflammatory processes and hyperplasia of the tissue favoring further inflammation. The sequelae of hyperplasia of the pharyngeal tonsils are among others an obstruction of the pharyngeal ostium of the Eustachian tube and a transition of the inflammation to the mucosa of the Eustachian tube leading to chronic tube dysfunction. Based on this tube dysfunction with compromised ventilation of the middle ear, otitis media with and without effusion develop [72].

#### 1.4.3 Predisposing factors

There are hints that a cytokine polymorphism represents a genetically predisposing origin of acute otitis media. Investigations allow the assumption that polymorphism of the tumor necrosis factor alpha (-308) as well as interleukin-6 (-174) is associated with an increased risk of developing otitis media. This risk can be influenced by environmental factors such as e.g. breast feeding [88]. It became obvious that those two factors increase the probability to acquire otitis media after infection of the upper respiratory tract [98]. Furthermore, there are hints that polymorphism of interleukin-10 (IL-10) also favors the occurrence of otitis media [2]. Probably, genetic polymorphisms of the cytokines TNFA-863A, TNFA-376G, TNFA-238G, IL-10-1082A, and IL-6-174G may lead to an increased occurrence of acute otitis media [35]. Investigations on single nucleotide polymorphisms revealed an increased infection risk for IL-1β (-31), CX3CR1 (Thr280Met), IL-10 (-1082) as well as IL-1β (-511) [80]. 

Casselbrant et al. found a genetic proof of the incidence of otitis media: investigations of 168 twins and 7 triplets over a period of 13 years revealed that a genetic component contributes to the incidence of acute otitis media with and without effusion. Thus high-risk patients should be examined in narrow intervals [20].

#### 1.4.4 Others 

There are hints that weight loss induced by bariatric surgery may lead to an increased occurrence of tube dysfunction [87]. Also cranio-facial deformities such as cleft lip and palate may be associated with tube dysfunction [106].

## 2 Clinic of otitis media

### 2.1 Serous otitis media 

Persisting tube ventilation disorder and resulting negative pressure in the middle ear lead to an increased secretion of the middle ear epithelium. This is the origin of serous otitis media which may occur as serotympanum or mucotympanum. The secretion is initially serous. In the further course of the ventilation disorder, an increasingly mucous consistency is observed, the so-called glue ear. Clinically, the eardrum appears mostly matt and sometimes amber [64].

### 2.2 Acute otitis media

**Figure 8:**
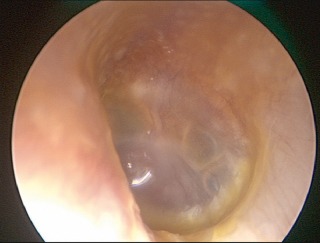
Endoscopic view (0° optic) of beginning otitis media of the left middle ear. The typical aspect of beginning radial vascular drawing, injection of the vessels of the malleus handle, effusion with reddening and beginning protrusion of the tympanic membrane as well as cloudiness of the surface of the eardrum are found.

**Figure 9:**
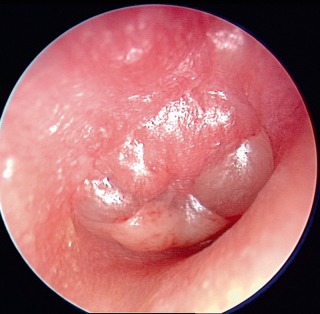
Endoscopic view (0° optic) of an advanced acute otitis media of the right middle ear. Severe inflammatory reaction and protrusion of the eardrum are found (source and copyright: Lenarz T, Boenninghaus HG. HNO. 14th Edition, 2012, page 87, [64]. Courtesy of Springer).

Significant clinical characteristic of acute otitis media are a reduced transparency and hypomotility of the eardrum [57]. The appearance varies considerably and can be categorized according to parameters such as position, color, transparency, or motility of the eardrum. In the context of the clinical picture of acute otitis media, protrusion of the eardrum is found in around 28% of the cases, mild protrusion in 45%, and in 19% no protrusion is observed. Perforation of the eardrum is found in 6%. The inflammatory reaction of the eardrum is mild in 6%, moderate in 59%, and severe in 35% of the cases [56] (Figure 8 (Fig. 8) and Figure 9 (Fig. 9)).

Earaches are the main symptom of acute otitis media [47], [79], often even combined with fever [52]. But in children also sore throat or sleeplessness are considered as hints [58].

Because of the incompletely matured defense, the still thickened mucosa of the antrum and the tympanum, and the short and relatively wide tube in infants, otitis may occur more easily and develop severely [4]. Furthermore, acute otitis media in infants may lead to failure to thrive beside other complications [61].

### 2.3 Chronic otitis media

**Figure 10:**
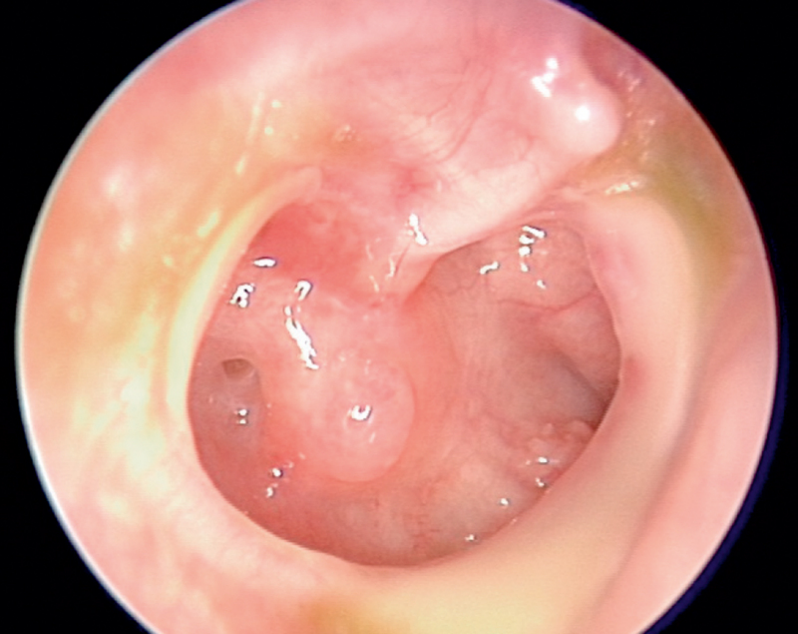
Endoscopic view (0° optic) of chronic otitis media of the right middle ear. Mesotympanic defect of the tympanic membrane with preserved fibrous ring is found. The mucosa of the tympanic cavity is swollen and secreting (source and copyright: Lenarz T, Boenninghaus HG. HNO. 14th Edition, 2012, page 94, [64]. Courtesy of Springer).

Chronic otitis media is categorized into mesotympanic chronic otitis media and epitympanic chronic otitis media. In cases of mesotympanic chronic otitis media, a mesotympanic defect of the tympanic membrane is found, the annulus fibrosus is intact. Often an inflammatory and humid mucosa of the middle ear is found (Figure 10 (Fig. 10)), but also dry courses with pale-grey tympanic mucosa are observed. Epitympanic chronic otitis media (cholesteatoma) is characterized by a marginal defect with the clinical appearance of a sometimes even year-long smelling suppuration [64]. Chronic otitis media is associated with a more or less developed conductive hearing loss.

## 3 Diagnostic procedures

### 3.1 Otoscopy

The most important diagnostic measure is otoscopy. There are several techniques for this purpose. For many years, the examination with a head mirror (circular concave mirror with a small hole in the middle, focal length of 10–20 cm) was common practice. Nowadays, the use of ear microscopes is established that magnify 6–40 fold. Furthermore, endoscopes are applied. Alternatively also head lamps and otoscopes may be used [64].

### 3.2 Audiometry

Pure tone audiometry is considered as the basic diagnostic procedure. Air-bone gaps can be detected in the context of inflammatory diseases of the middle ear and also in the context of tube dysfunctions. Also speech audiometric measurements or further audiological diagnostic procedures can be applied to confirm of the diagnosis [64].

### 3.3 Tympanometry

As diagnostic procedure to measure the ventilation of the middle ear, tympanometry is established. Hereby the pressure-depending variation of the impedance can be registered by measuring the reflected portion of the probe sound in form of a graph. Based on positive pressure, equal pressure, and negative pressure, the portion of the reflected probe sound can be measured and registered as graphic presentation. In this way, information on the middle ear pressure is gained. In cases of middle ear catarrh or otitis media with effusion, often flat graphs are found that may even be shifted to the left. In the latter case, also flat graphs with missing proof of stapedius reflexes may be registered [64].

### 3.4 Diagnostics of tube function

In order to assess the function of the Eustachian tube, a tube score was developed. It consists of the feasibility of Valsalva’s maneuver (yes/sometimes/no, with respective scores of 2/1/0 points), the possibility of pressure compensation by swallowing (yes/sometimes/no with respective scores of 2/1/0 points) as well as R value of tube manometry with 30, 40, and 50 mbar. The R value is the opening latency index and is defined by the time between pressure application in the nasopharynx and measurement of pressure alteration in the auditory canal. R values of ≤ 1/> 1/no R value or no tube opening correspond to 2/1/0 points in the tube score. In summary, the tube score may achieve results from 0 to 10 points. Hereby, 0 points correspond to severely obstructive tube dysfunction and 10 points reveal and optimal tube function [110] (Table 3 (Tab. 3)). Especially tube manometry develops as an established diagnostic procedure to support the diagnosis of chronic obstructive dysfunction of the Eustachian tube [107].

## 4 Evidence-based therapeutic approaches

### 4.1 Historical facts

Therapeutic approaches of the 19^th^ century regarding impaired middle ear ventilation and otitis media were not evidence-based. They consisted for example in the application of a water or air rinsing, the introduction of a fine harp string into the tube, even against resistances that were overcome by enhanced pressure. Other therapeutic approaches were the use of leeches, avoiding spicy food, drinking hot beverages, or the recommendation of mucus producing food and beverages. Furthermore, eating butter, cheese, milk and milk products, legumes, and sour and fatty food of all types was expected to help. Water, light bitter beer, red wine with water, strong lean meat were also recommended as well as intensive physical moving and possibly waking up early in the morning. The repeated administration of emetics was not recommended. It was also common opinion that abundant comfortable defecation was certainly a patient’s need but generally could not be considered as benefit for the mucosa of the pharynx. Furthermore, it turned out to be without success to apply gargling, Spanish flies, fontanelles, hair cords, suppuration with tartar emetics, continuous irritation of the skin of the neck with small galvanic devices from a copper and a zinc plate. Unsuccessful therapeutic attempts were undertaken with bougie and sponges [59].

Major contributions to the development of therapeutic procedures were made among others by Joseph Toynbee and Adam Politzer.

Joseph Toynbee was born in England in 1815. He was an English physician and otologist. Among others, he developed the procedure that is known today as Toynbee maneuver being applied today for diagnosis of tube dysfunction. The Toynbee maneuver consist of the procedure that the patient causes negative pressure in the middle ear by holding his nose and swallowing at the same time. A physiological inward movement of the tympanic membrane takes place which cannot be observed in cases of tube dysfunction [94]. 

Adam Politzer was born in Hungary in 1835. Since 1861 he was lecturer of otology at the University of Vienna. In 1871 he was appointed extraordinary professor and together with Josef Gruber he organized the Ear Hospital of Vienna since 1873. He developed the procedure that was named after him consisting of active ventilation of the middle ear by blowing air up the nose with a rubber air-bag during the act of swallowing, which closes the palate. Thus he was able to compensate the negative pressure in the middle ear in cases of tube ventilation disorders by positively influencing the tube function [132]. 

At the beginning of the 20^th^ century, another historical reflection regarding the treatment of tube dysfunction consisted in the local application of thorotrast. Thorotrast was the brand name of a radio contrast agent that was introduced in the market in 1929 and based on a stabilized colloidal 25% suspension of thorium dioxide. Because of the radioactivity of thorium, the topical application was expected to have positive effects on the mucosa of the Eustachian tube. However, the colloidal thorium dioxide accumulated in the reticuloendothelial system. Correlations with the incidence of cholangiocarcinomas, angiosarcomas, hepatic hemangio-endotheliomas, or carcinomas of the paranasal sinuses were described. Typically, the diseases developed 30–35 years after exposition [60], [117]. That is why the application of thorotrast could not be established, furthermore there were no studies regarding the application in the context of tube dysfunction. 

### 4.2 Evidence-based therapeutic approaches of tube dysfunction

#### 4.2.1 Tube wire

The idea of using tube wires are based on Politzer’s therapeutic approaches with tube catheters. Politzer applied them as catheterization of the Eustachian tube in patients who had undergone unsuccessful air rinsing. Hereby he used elastic, well-polished and easily sliding hard rubber catheters or alternatively new silver catheters that were introduced through the nose into the Eustachian tube [132]. Based on this procedure, the treatment of tube dysfunction with tube wire was a therapeutic option. This wire is inserted into the tube in order to secure patency. Studies from the 1990s report that this method achieved significant improvement of middle ear ventilation after follow-up intervals of 2–50 months in 11/13 patients [69]. 

A retrospective study evaluating 96 patients from 1996 to 1999 who were followed-up for 18 months revealed that the transtympanic implantation of golden tube wire led to postoperative normalization of the tube function in 8 patients (8.3%). However, in 88 patients (91.7%) the tube dysfunction did not change. Successively, the tube wires were removed because of the persisting ventilation disorder, dislocations, or granulation around the tube wire. So this therapy of tube dysfunction was considered as not being appropriate [111].

Because of the study situation, the evidence of this obsolete therapeutic approach can be classified as grade C according to the GRADE classification (Table 4 (Tab. 4)).

#### 4.2.2 Balloon dilation of the Eustachian tube

**Figure 11:**
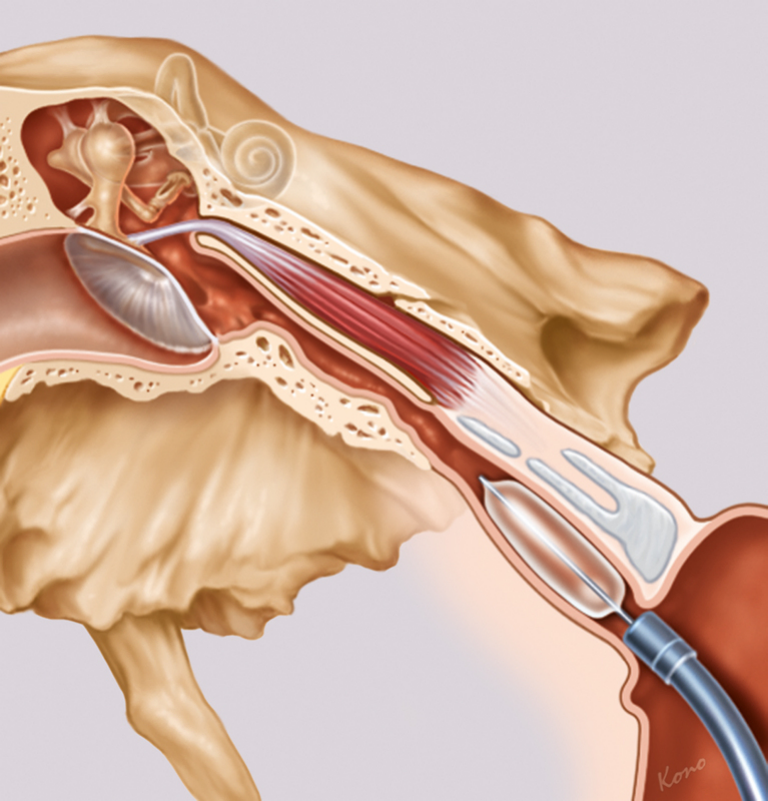
Balloon catheter for tube dilation. Tube dilation by means of balloon catheter provides new possibilities to treat tube dysfunction (courtesy of Spiggle & Theis, Overath, Germany).

Nowadays, the therapeutic approaches are better reflected. To improve the tube function in cases of dysfunction a procedure of balloon dilatation of the Eustachian tube was developed (Figure 11 (Fig. 11)). First applications of this procedure were described in 2009 [82]. Published case studies from 2010 show that balloon dilation was a feasible and safe procedure [83], [84]. Retrospective assessments of 30 patients with the Glasgow Benefit Inventory revealed significant improvement of psychosocial, social, and general health factors 6–18 months after tube dilation [9].

Regarding short-term results of tube dilation, first publications are meanwhile present. Retrospective clinical assessments on short-term results in 21 patients revealed subjective improvement in 76% of the patients, regular R values were achieved in 57% of the patients, and retractions of the tympanic membrane improved in 18% of the patients [44]. Additionally, the analyses of case studies confirm that the possibility of performing Valsalva maneuvers after tube dilation improved from 11% to 64% [76]. 

Evaluations of the tube scores of 66 patients (115 dilations) revealed that it improved significantly after 2 months from 2.21 (±2.02 SD) to 5.4 (±2.53 SD). Analyses of dilations of 12 patients (20 dilations) after 12 months showed an improvement from 1.25 (±1.83 SD) to 6.2 (±2.61 SD) [110].

In addition, the results of 622 patients after balloon dilation of the tube (1,072 ears) could be analyzed in the long-term course. One year after dilation, an improved tube score from 3.13 (±2.47 SD) to 5.75 (±2.76 SD) could be observed. In 73 of the treated ears, a significant improvement of the tube score was registered. Two years after balloon dilation, an improvement of the value from 2.65 (±2.89 SD) to 6.26 (±3.07 SD) could be achieved. First long-term studies showed good results after 5 years. In 82% of the patients the tube score was significantly improved. The subjective satisfaction was around 80% [107]. Even in children, balloon dilation is more and more often applied [54], [122].

But meta-analyses reveal that the evidence of tube dilation is not sufficiently confirmed until now. Certain conclusions are not possible and neither is the identification of patients who will benefit from balloon dilation. Furthermore, the results of this therapy cannot be predicted with sufficient probability [96]. Especially randomized controlled trials and further study results are currently not available. Furthermore, the effect of the reduction of the air-bone gap by this procedure which is most important for the patients is not evaluated. Future research will have a crucial influence on the estimation of the effect. Thus, the study situation has to be considered as highly limited and the grade of evidence of tube dilation must be classified as grade C. The currently initiated multicenter trials will contribute to further data regarding the evidence.

#### 4.2.3 Laser therapy

Laser surgical endoluminal approaches for the treatment of tube dysfunction were evaluated in prospective study approaches. Ten patients with chronic tube ventilation disorders were treated with 980 nm diode or argon laser. Hereby, parts of the mucosa and the cartilage of the posterior wall of the tube lumen were coagulated with the laser. In 5 patients, follow-up examinations could be performed after 12 months, the complaints of 3 patients had improved. However, there was no control group in this study [92]. Another prospective study investigated the effects of fiberoptic laser ablation of the posterior part of the tube ostium. Hereby an improvement of the tube function could be confirmed in 26 of 38 patients (68.4%, p=0.001). The effect persisted 1 year after the intervention [112]. In another study, 108 tube dysfunctions in 56 patients were evaluated after laser therapy with CO_2_ laser or 980 nm diode laser. The mucosa and the cartilage of the posterior wall of the tube were coagulated with the laser. Furthermore, laser myringotomy was performed to achieve temporary middle ear ventilation. The success of this measure was assessed by means of impedance tympanometry as well as the presence or absence of effusion or retractions of the tympanic membrane. In 68.5% of the ears treated in this way, a regular ventilation situation of the ears could be achieved 1 year after the intervention. After 2 years, the success rate was 71.4% and after 3 and more years it was 65.2%. Intraoperative complications were not described. Postoperative, synechia occurred in 8.3% of the cases [62]. However, the success is not only due to the laser treatment of the tube because myringotomy was performed at the same time. Furthermore, there was no control group.

Also regarding this therapeutic approach there are only studies with high limitations and it is most probable that future research results have a decisive impact on the estimation of the effect. So, also in this context the evidence level C must be assumed according to the GRADE classification (Table 4 (Tab. 4)).

### 4.3 Evidence-based therapeutic approaches of otitis media

#### 4.3.1 Therapy of pains

Otalgia is a frequent symptom of acute otitis media. Investigations revealed that otitis media with effusion is associated with severe otalgia in 42% and moderate otalgia in 40% of the patients. In 17% of the cases, no earaches were reported [46]. Generally, the pains persist for 3–7 days [101]. Therefore in cases of pains an adequate pain therapy should be performed. Several pharmaceutics are available for this treatment. None of those pharmaceutics, however, has yet been finally evaluated in the clinical context of otitis media. The treating physicians should always make the decision together with the patient based on weighing the advantages and risks of analgesics [68]. 

A combination of an analgesic with antibiotic application leads to optimized pain therapy. A placebo-controlled trial from 1991 [17] could show that the administration of amoxicillin 125 mg 3x per day for 7 days together with 100 ml paracetamol 120 mg/5 ml in comparison to paracetamol alone led to improved pain symptoms in children after a short interval. The mean time until the pains had disappeared was 2.82 days in the antibiotic group in comparison to 3.34 days in the placebo group.

The effectiveness of ibuprofen for the treatment of otalgia in the context of acute otitis media was evaluated in a randomized, placebo-controlled multicenter double-blind trial. 219 patients aged between 1 and 6 years could be included in this study. The patients either received 10 mg/kg ibuprofen (n=71), acetaminophen (paracetamol) (n=73), or a placebo (n=75) as oral treatment. All patients additionally received cefaclor for one week. Regarding the treatment of the pains, it could be revealed that ibuprofen achieved a significant reduction of the pains. For acetaminophen (paracetamol), also pain reduction was observed, which, however, was not significant. With regard to the course of the inflammation, no significant difference was observed in the groups compared to the placebo group [11]. 

Another double-blind study evaluated the effectiveness of 2% lidocaine ear drops in comparison to topical saline solution application in children between 2 and 17 years. A clear effectiveness with significant improvement of the pain values could be shown 10 minutes after application of the analgesics [13].

Also an evaluation of the effectiveness of topical application of a mixture of antipyrine, benzocaine, and glycerin revealed improved pain symptoms, however, with significance only after an interval of 30 minutes [49]. Evaluations on the effectiveness of procaine and phenazone in children with otitis media showed a clear reduction of the pains in 93% of the cases [1].

In summary, there are several high-quality studies with consistent results, but they assess different substances and combinations. Thus future research results will have an important effect on the estimation of the effect and it is possible that the effect might change. The evidence is classified as grade B (Table 4 (Tab. 4)).

#### 4.3.2 Antibiotic therapy

Antibiotic therapy is considered as therapy of choice for acute otitis media. Since most pathogens causing acute otitis media in children are organisms producing beta-lactamase, a high-dose therapy with amoxicillin preparations are the treatment of first choice followed by other beta-lactamase stable pharmaceutics [91].

The evidence of antibiotic therapy was confirmed by numerous trials that were published. A randomized placebo-controlled double-blind study of 84 children between the ages of 6 months and 15 years showed that children receiving antibiotic therapy with amoxicillin clavulanic acid in a dosage of 40 mg/kg experience quicker healing of otitis media than without this therapy. In the median, healing was experienced after 2.7 weeks, defined as regular tympanogram on 2 subsequent days. In the control group, healing was observed only after 4.7 weeks. Furthermore, inconspicuous otoscopic findings were found in the study group with antibiotics 1.4 weeks earlier than in the placebo group [118].

The effectiveness of antibiotic treatment of children aged between 6 and 35 months suffering from acute otitis media was confirmed in a randomized, placebo-controlled trial by Tähtinen. It could be shown that therapy failure occurred in 18.6% of the patients receiving amoxicillin/clavulanic acid, but in 44.9% of the patients of the placebo group. The difference between both groups was obvious already 3 days after therapy onset. Therapy with amoxicillin/clavulanic acid reduced the progress of the disease by 62% and the necessity of so-called rescue therapy by 81%. However, diarrhea was observed as side effect of the application of amoxicillin/clavulanic acid in 47.8% which was significantly higher than in the placebo group with 26.6%. Also the incidence of eczema was higher in the study group with 8.7% compared to the placebo group with 3.2% [119].

Another placebo-controlled trial with children aged between 6 and 23 months with acute otitis media revealed that the treatment with amoxicillin/clavulanic acid for 10 days reduced the duration of the clinical symptoms of the disease, reduced the distress caused by the symptoms, and reduced the rate of persisting signs of acute infection. However, no significant changes were observed in both groups regarding the nasopharyngeal colonization with Stre*ptococcus pneumoniae* [51].

Another study investigated the effectiveness of amoxicillin and clavulanic acid. This study could include 79 children in the treatment group and 70 in the placebo group. Patients of the study group received 20 mg/kg amoxicillin and 5 mg/kg clavulanic acid for 14 days. The patients of both groups additionally received xylometazoline 0.25% as topical nasal application 3 times per day. In the follow-up, patients of the study group had a clearly lower persistence of the complaints (53 vs. 84%) than the control group (77 vs. 93%). Also here, clearly more side effects were observed in the study group than in the control group (44 vs. 22%, p=0.03). Side effects were mostly gastrointestinal complaints with a mild course [128].

If the portion of clavulanic acid is changed to a ratio of 1:7, the incidence of diarrhea decreases. Evaluations of 868 children aged between 2 months and 12 years revealed that combined administration of amoxicillin and clavulanic acid in the mentioned ratio (amoxicillin:clavulanic acid:45:6.4 mg/kg/day) for 10 days reduced the effectiveness of the therapy and the incidence of diarrhea compared to a higher dosed part of clavulanic acid of 1:4. An application for 5 days also showed a lower effectiveness in comparison to an administration for 10 days [49].

Also other studies confirm the recommendations of antibiotic therapy for 10 days. In prospective double-blind randomized multicenter studies, therapy for 10 days led to a higher healing rate compared to therapy for 5 days [27]. However, 4–6 weeks after therapy, no differences between both study groups could be found [26]. Other studies see the advantage of therapy for 10 days only in cases of recurrent otitis episodes [90].

Further meta-analyses revealed that antibiotic therapy in children younger than 2 years with bilateral acute otitis media as well as children with acute otitis media and otorrhea is effective and can be recommended. For children with mild symptoms, however, first only clinical control seems to be justified [100]. Prophylactic antibiotic therapy in cases of recurrent acute otitis media is not recommended [68]. 

The study situation also shows data that do not confirm a significant effect of antibiotic therapy: a double-blind study from 1981 compared the therapy results of acute otitis media of 171 children receiving neither antibiotic nor myringotomy, only myringotomy, only antibiotics, and myringotomy and antibiotics. No significant differences of the clinical courses were found regarding pain symptoms, temperature, duration of the complaints, otoscopic findings, audiometric findings, or recurrence rates between those four groups. In the groups without antibiotic treatment, healing took more time but the differences were not significant. Complications were not observed [129]. Based on the described study situation, however, this last mentioned trial can be neglected in an evaluation.

Thus, in Germany the indication of immediate antibiotic therapy is recommended in cases of acute otitis media. Thomas et al. recommend the application according to the following scheme considering the age and other factors (based on the Oxford evidence level and on good clinical practice (GCP)): age <6 months (evidence level Ia; GCP: A); age <2 years with bilateral acute otitis media, mild earaches and temperature <39°C (evidence level Ia; GCP: A); acute otitis media with moderate to severe earaches or temperature ≥39.0°C (evidence level Ib: GCP: A); persisting purulent otorrhea (evidence level Ia; GCP: A); presence of risk factors (e.g. otogenic complications, immune deficiency, severe basic diseases, Down syndrome, cleft lip and palate, cochlear implantation, influenza) (evidence level III, GCP: B), or in the case that follow-up within the first 3 days is not possible (evidence level Ib; GCP: A) [121].

The recent recommendations from the USA (American Academy of Pediatrics) consists of first-line therapy with amoxicillin (80–90 mg/kg per day in 2 doses) or amoxicillin combined with clavulanic acid. Alternatively, for example in cases of penicillin intolerance, cephalosporins such as cefuroxime (30 mg/kg per day in 2 single doses) or also cefpodoxim (10 mg/kg per day in 2 single doses) are available. Amoxicillin/clavulanic acid (90 mg amoxicillin/kg per day) combined with 6.4 mg clavulanic acid/kg per day in 2 single doses or also ceftriaxone (50 mg i.m. or i.v. for 3 days may be applied as antibiotic therapy after 48–72 hours or in cases of treatment failure. Alternatively clindamycin in a dosage of 30–40 mg/kg per day in 3 single doses may be administered, also combined with ceftriaxone or cephalosporins (Table 5 (Tab. 5)) [68]. Even in cases of serotympanum, antibiotic therapy is effective [3], [71]. The complication rate under conservative antibiotic therapy is low and stable with around 2%.

In summary, there are several high-quality studies with consistent results. It is rather improbable that future research will change the estimation of the effect. Thus the evidence of antibiotic therapy for otitis media is classified as GRADE A (Table 4 (Tab. 4)).

#### 4.3.3 Adenoidectomy

Adenoidectomy combined with tonsillectomy reduces the charge of potential pathogens in the pharynx and thus counteracts an inflammation reaction [63], [73]. It is indicated in cases of adenoid hypertrophy associated with nasal obstruction, in cases of recurrent otitis media as well as chronic otitis media, tube ventilation disorder with seromucotympanum, rhonchopathy, or mastoiditis as consequence of chronic tube ventilation disorder [31], [32], [95]. Even severe diseases of the airways justify adenoidectomy [16]. In healthy, asymptomatic children with nasal obstruction or chronic inflammation of the adenoids, however, adenoidectomy is not recommended [99].

Meta-analyses of independent randomized studies confirm the effectiveness of adenoidectomy as surgical treatment option in children with persisting otitis media with effusion and condition after ineffective conservative therapy. Hereby, a significant reduction of the morbidities is observed after adenoidectomy in comparison to control groups [39]. Further meta-analyses also confirm that adenoidectomy in children leads to significant reduction of rhinosinusitis complaints [14]. Furthermore, a significant reduction of obstructive sleep apnea phases is confirmed after adenoidectomy [16], [76], [114], [124], [125].

Even long-term evaluations of 110 patients aged between 2 and 12 years reveal a significant effectivity regarding inflammatory consequences up to 7 years after adenoidectomy with tonsillectomy [36].

Meta-analyses further show that 2 subgroups of children benefit most from adenoidectomy: on the one hand those are children with recurrent acute otitis media younger than 2 years. Only in 16% of the patients therapy failure was observed after 12 months, in the control group without adenoidectomy this value amounted to 27%. On the other hand, children older than 4 years who suffer from chronic otitis media with effusion benefit from adenoidectomy. Hereby, therapy failure was observed in 51% of the patients after 12 months compared to 70% in the control group without adenoidectomy. No significant advantage of adenoidectomy could be observed in children older than 2 years with recurrent otitis media and children younger than 4 years with chronic otitis media with effusion [14].

But some studies also show that adenoidectomy with or without ventilation tubes did not provide an advantage for small children with chronic otitis media with effusion in comparison to children who received only ventilation tubes [21]. In summary, numerous high-quality studies are available with consistent results that justify a classification of evidence GRADE A (Table 4 (Tab. 4)).

#### 4.3.4 Paracentesis and tympanostomy tubes

**Figure 12:**
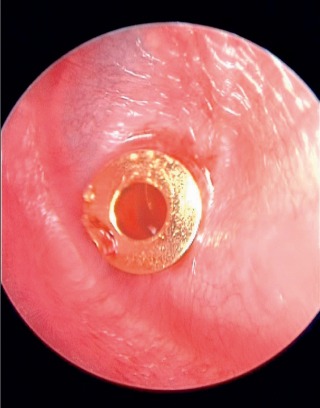
Endoscopic view of gold ventilation tube inserted in the eardrum (source and copyright: Lenarz T, Boenninghaus HG. HNO. 14th Edition, 2012, page 84, [64]. Courtesy of Springer).

The insertion of ventilation tubes (Figure 12 (Fig. 12)) is considered as effective therapy of chronic otitis media with effusion [95]. In cases of acute otitis media, myringotomy is indicated for persisting fever, pains, and protrusion of the tympanic membrane without perforation as well as beginning complications such as irritation of the labyrinth, weakness of the facial nerve or meningism. Also the occurrence of advanced complications such as mastoiditis, labyrinthitis, or meningitis justifies myringotomy. The insertion of tympanostomy tubes may complete the intervention [64]. Surveys from Canada showed that there is no consensus regarding the indication of tympanostomy tubes. 40% of the ENT physicians replied that they never insert tympanostomy tubes, 30% sometimes perform this intervention, and 30% often perform this intervention [74].

The effect of myringotomy [129], [131] or insertion of tympanostomy tubes [86] has been discussed controversially for many years. However, review articles clearly show advantages of ventilation tubes compared to myringotomy alone in patients with otitis media with effusion persisting for more than 2–3 months [33]. Other studies confirm a significant reduction of the number of acute otitis media episodes after insertion of ventilation tubes during a 6 months follow-up [41], [42]. Again other studies found no effect of insertion of ventilation tubes during a follow-up interval of 2 years [19]. A prospective multicenter cohort study on the outcome of ventilation tubes in children with otitis media, however, revealed that therapy of otitis media with ventilation tubes significantly improves the quality of life – measured among others with audiometric and psychosocial parameters [135].

Thus it is recommended in cases of recurrent otitis media (3 episodes within 6 months or 4 episodes in one year) to insert ventilation tubes [68].

In summary, the availability of several high-quality studies with almost consistent results justified classification of the evidence of myringotomy and insertion of ventilation tubes with GRADE A (Table 4 (Tab. 4)).

#### 4.3.5 Antiviral therapy

Impaired ventilation of the middle ear with subsequent acute otitis media is often caused by viral pathogens. In 30%, infections of the respiratory tract lead to acute otitis media and in about 8% to sinusitis. Around 10% of the children with acute otitis media do not show any hint for bacterial genesis [97]. Therapeutic approaches with antiviral agents, as for example discussed for therapy of influenza infection (in this context antivirals of the first generation (M2 blocker, amantadine/rimantadine) or antivirals of the second generation (neuramidase inhibitors: oseltamivir, zanamivir) are discussed) are generally not applied in the therapy of tube dysfunction, acute otitis media, or otitis media with effusion. Further development of antiviral therapeutic concepts might lead to new treatment strategies in the future [134]. An effective vaccination against infections of the upper respiratory tract caused by respiratory syncytial virus, for example, might possibly reduce the incidence of acute otitis media [48].

#### 4.3.6 α-sympathomimetic agents

The effect of topical nasal application of α-sympathomimetic agents for therapy of otitis media was investigated in several studies.

In a randomized, double-blind, placebo-controlled study of children with persisting otitis media, the tube function was measured before and after intranasal application of 5 drops of 0.05% xylometazoline hydrochloride or placebo in 80 randomly selected children with ventilation tubes because of otitis media with effusion. No effect was observed on the ventilation function or the protective function of the Eustachian tube. Thus no positive effect on the tube function in children could be confirmed regarding topically applied, decongesting agents [130].

Other authors of a randomized, double-blind, placebo-controlled study of 36 patients aged between 12 and 75 years found a positive effect of xylometazoline 0.1% only after direct application on the torus tubarius and only after application of unphysiologically high pressure in the context of Valsalva’s maneuvers, but not after testing with lower pressure [55].

In another randomized, placebo-controlled, double-blind study, the effect of topically applied nasal decongesting sprays on the function of the Eustachian tube, 40 children with severe tube dysfunction and tympanostomy tubes were evaluated. Hereby either oxymetazoline or placebo were applied nasally. Also here, no significant difference could be revealed between both treatment groups [70].

Thus the application of topical, decongesting agents to avoid or treat serous otitis media is considered as being ineffective [99].

Even in cases of acute otitis media, meta-analyses reveal no positive effect of decongesting pharmaceutics on the healing rates. The symptoms of acute otitis media do not decrease more rapidly, no lower number of surgical indications is observed, neither is a lower complication rate [29]. In summary, several high-quality studies are available with consistent results and it seems rather improbable that future research results change the estimation of the effect. This means that the evidence of this therapeutic approach is GRADE A, however, with an evidence of the non-effectiveness. This aspect is in contrast with established clinical practice performed since many years in an experience-based way.

#### 4.3.7 Steroids

Studies could not confirm the effectiveness of corticosteroids for therapy of tube dysfunction and otitis media and are not recommended for treatment [99]. Further effects are not expected by other studies so that this treatment is classified as GRADE A (Table 4 (Tab. 4)).

#### 4.3.8 Therapy of chronic otitis media

Therapy of chronic otitis media mostly consists of surgical treatment [64]. In this context, numerous approaches and techniques are available that cannot be classified regarding their evidence because of their multitude. For every procedure, the study situation would have to be evaluated and undergo separate classification. Furthermore, the surgical skills and the experience of the surgeon is crucial for the outcome of the treatment so that classification with an evidence grade is not made in this contribution.

## 5 Evidence gaps

For therapy of compromised middle ear ventilation and otitis media, therapeutic approaches are applied that should undergo evidence assessment regarding their effectiveness. In the following paragraphs some approaches are discussed exemplarily.

### 5.1 Long-term results of tube dilation

In Germany, the procedure of tube dilation has been established only since a short time. So, as already described, no long-term results of this treatment are available. Furthermore, there are no blind studies nor randomized study results. Trials with this regard are currently prepared (Table 4 (Tab. 4)).

### 5.2 Therapy of cleft tube

In cases of cleft tube, an augmentation of the torus tubarius can be performed with different materials, which is expected to avoid opening when the pressure is too low. First descriptions of injections with collagen were published in 1988 [75]. Another possibility is the use of a polydimethylsiloxane elastomer dissolved in a hydrogel of polyvinylpyrrolidone (VOX^®^ Implants). Investigations could show that after the first injection posterior and superior-anterior of the torus tubarius 39% of the patients experience significant improvement, 33% had mild improvement, and 28% observed no improvement of their complaints. So in two third of the patient, multiple injection was required. However, after the follow-up interval of one year two third of the patients were satisfied with the result of augmentation [107]. In summary, the study situation for this procedure is rather limited. An estimation of the effect is very uncertain. Thus, the evidence of the therapeutic option is classified as GRADE D (Table 4 (Tab. 4)).

### 5.3 Medicinal plants

The application of herbal products for therapy of infections of the upper respiratory tract is often postulated. Mostly those are inhalative applications that are recommended. Often the inhalation of eucalyptus preparations is recommended. But also products with anise, mint, houseleek, elder, coltsfoot, garlic, lavender, yarrow, ribwort, violets, spruce, chamomile, pine, horseradish, sage, thyme, cajeput, or myrtle are mentioned in the context of treatment of acute otitis media with or without effusion. The evidence regarding those therapeutics is rather low. Some studies report about a significant reduction of the clinical symptoms or improvement of the clinical picture [137]. Other trials report about improved otalgia [104].

However, each estimation of the effect is very uncertain. Based on the limited study situation, the evidence of those therapeutic options can only be classified as GRADE D (Table 4 (Tab. 4)). 

### 5.4 Homeopathic drugs

Controlled studies on the effectiveness of homeopathic agents in the context of treatment of acute otitis media are currently not available [7]. First pilot studies revealed that therapeutic success may be achieved but further studies with higher patient numbers are necessary in order to confirm the effectiveness [53]. An estimation of the effect is very uncertain. Because of the limited study situation, the evidence for this therapeutic option can only be classified as GRADE D (Table 4 (Tab. 4)).

### 5.5 Tube stent

**Figure 13:**
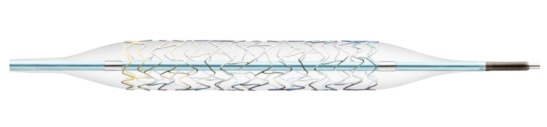
Presentation of a cobalt chrome stent (PRO Kinetic Energy. Courtesy of Biotronik Vertriebs GmbH & Co. KG, Berlin, Germany).

**Figure 14:**
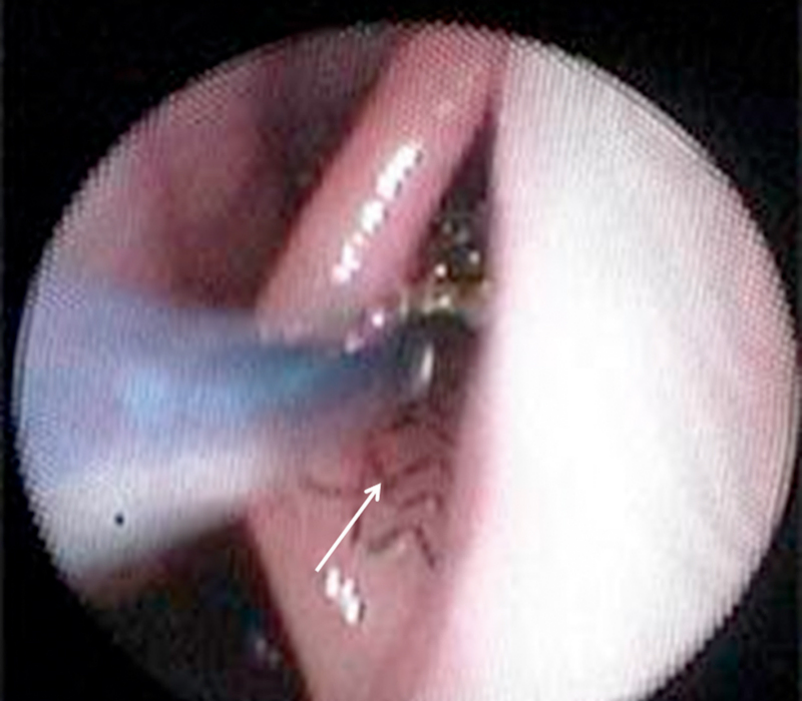
Endoscopic presentation of the tube ostium of a sheep with a stent inserted into the tube (arrow) (courtesy of F. Pohl, G. Paasche, R. Schuon, Medizinische Hochschule Hannover, Germany).

**Figure 15:**
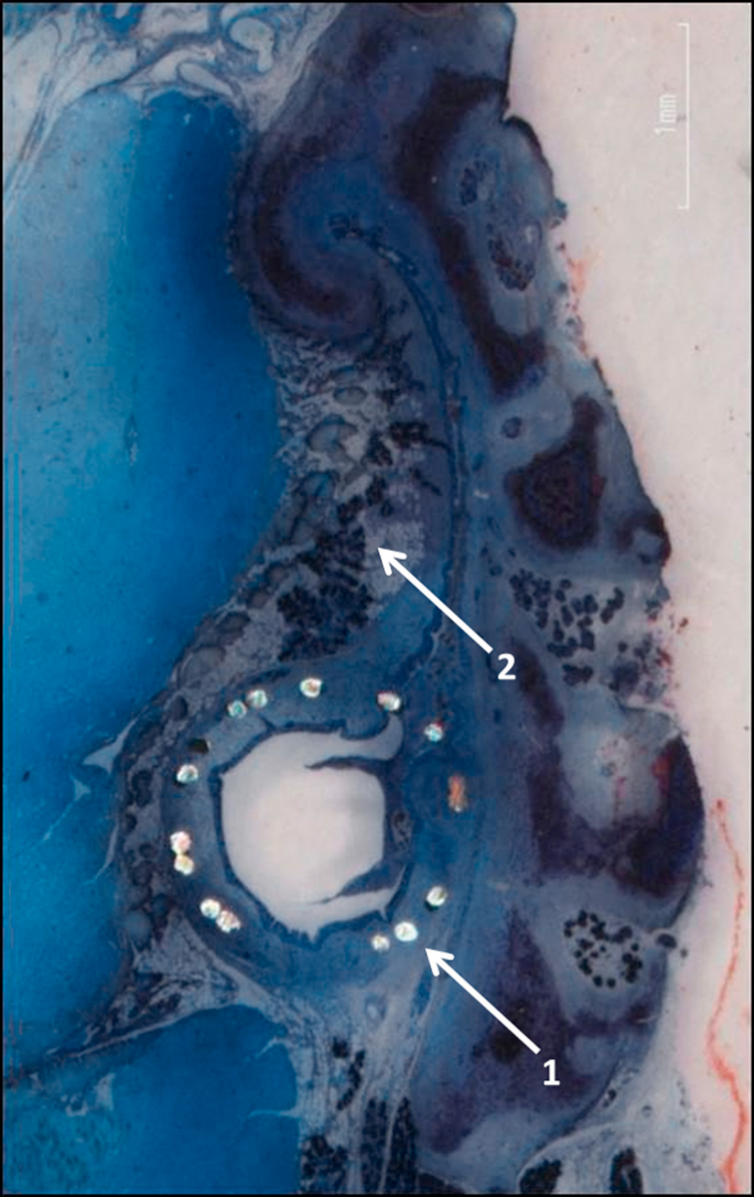
Histological examination (alizarin red – methylene blue in methylacrylate) of an Eustachian tube of a sheep with inserted stent (stent struts, arrow 1) with surrounding cartilage (arrow 2) (courtesy of F. Pohl, G. Paasche, R. Schuon, Medizinische Hochschule Hannover, Germany).

First trials for therapy of tube dysfunction by means of stents encompassed the use of a polyvinylchloride tube for enlargement of the tube. It had a thread and remained in situ for 10 days [138]. Later, trials were made with a tube stent. Hereby, good results could be reported with re-established ventilation of the middle ear in 79% of the cases. The tube stent that was used at that time was inserted through the tympanum [136]. However, the success rate could not be confirmed in a following study [65]. Currently tube stents are developed to improve the tube function with transpharyngeal insertion [77]. In first investigations with sheep, chrome cobalt coronary stents (ProKinetic Energy) (Figure 13 (Fig. 13)) were inserted into the tube (Figure 14 (Fig. 14)). The approach was performed endoscopically through the nose via the pharyngeal tube ostium. The insertion was without complications, neither bleeding nor lesions of the nasopharyngeal mucosa was observed. Histological examination showed regular positioning (Figure 15 (Fig. 15)) [93]. In summary, this therapeutic approach is still in the pre-clinical phase so that evidence-based recommendations are not possible.

## 6 Conclusion and outlook

For treatment of disturbed middle ear ventilation and otitis media, numerous evidence-based therapeutic approaches are available. Regarding several other options, the evidence situation is currently not sufficient or even recommendations against a therapy are given despite the fact that they were applied as clinically established and routine intervention for many years. The evidence gaps have to be closed in the following years. For this purpose, clinical studies are required in order to generate an evidence-based study situation. But also new therapeutic concepts as for example the use of tube stents have to be evaluated after clinical introduction with regard to their evidence. The necessity justifies the claim to conduct clinical studies; despite increasingly economic limitations and few resources, they will have to find excellent conditions in Germany.

## Notes

### Competing interests

The author declares the following conflict of interest: Reimbursement of travel expenses for 2 specific meetings and reception of an honorary by Spiggle & Theis Company, Overath, Germany.

## Figures and Tables

**Table 1:**
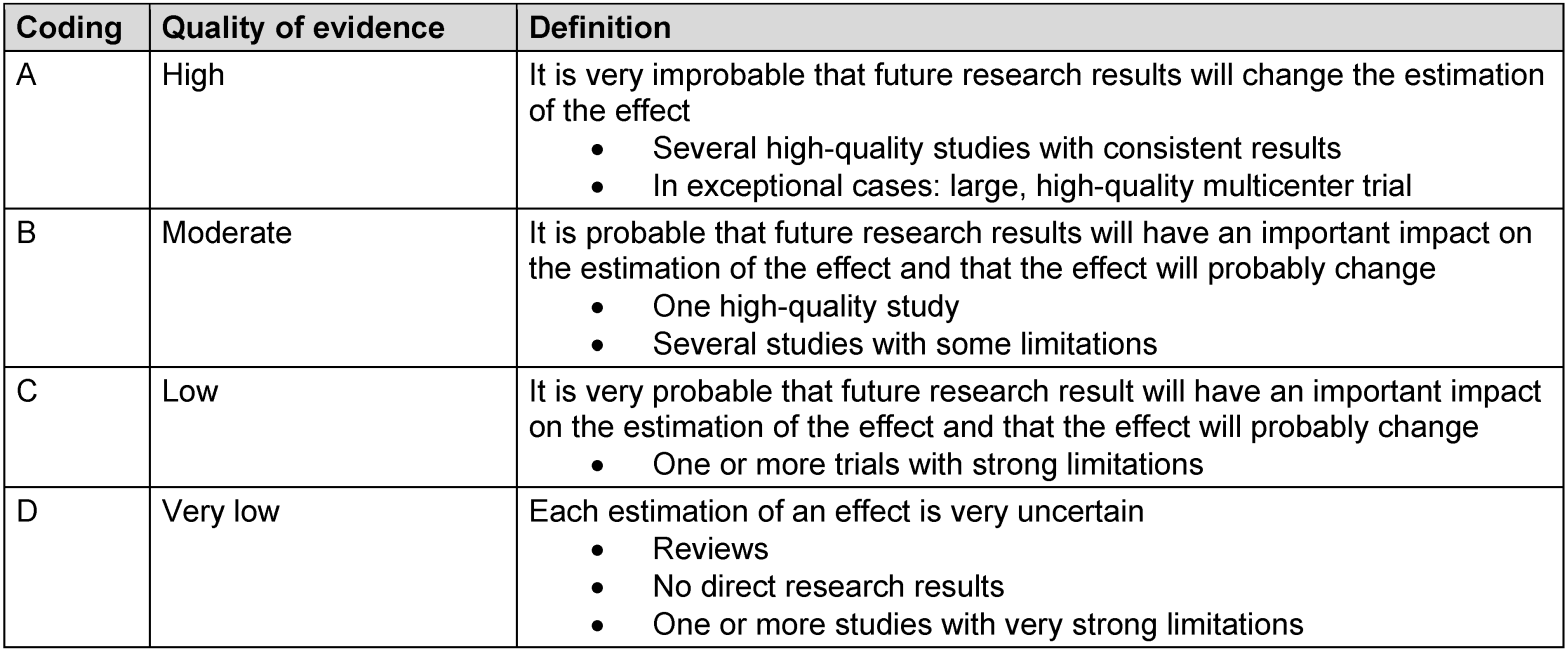
GRADE classification of the Grade Working Group on the classification of evidence [5, 38, 43, 45]

**Table 2:**
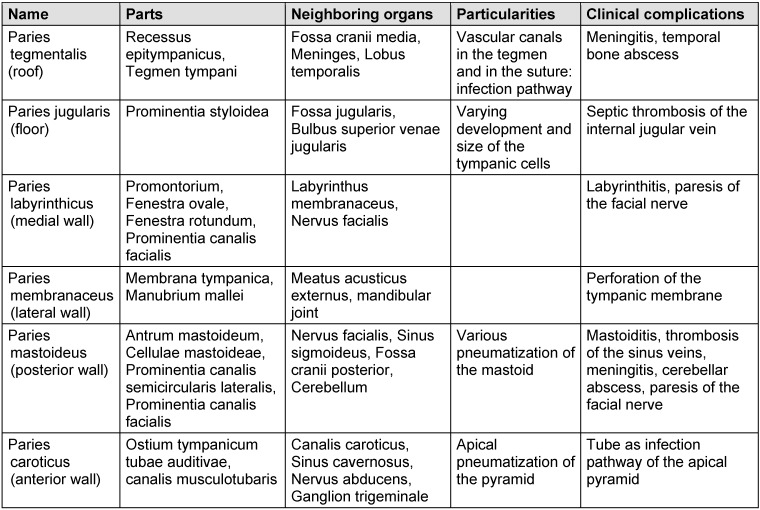
Anatomical limitations of the tympanic cavity (modified based on Sobotta. Atlas der Anatomie des Menschen. München, Wien, Baltimore. Urban & Schwarzenberg, 1993, page 371, [115])

**Table 3:**
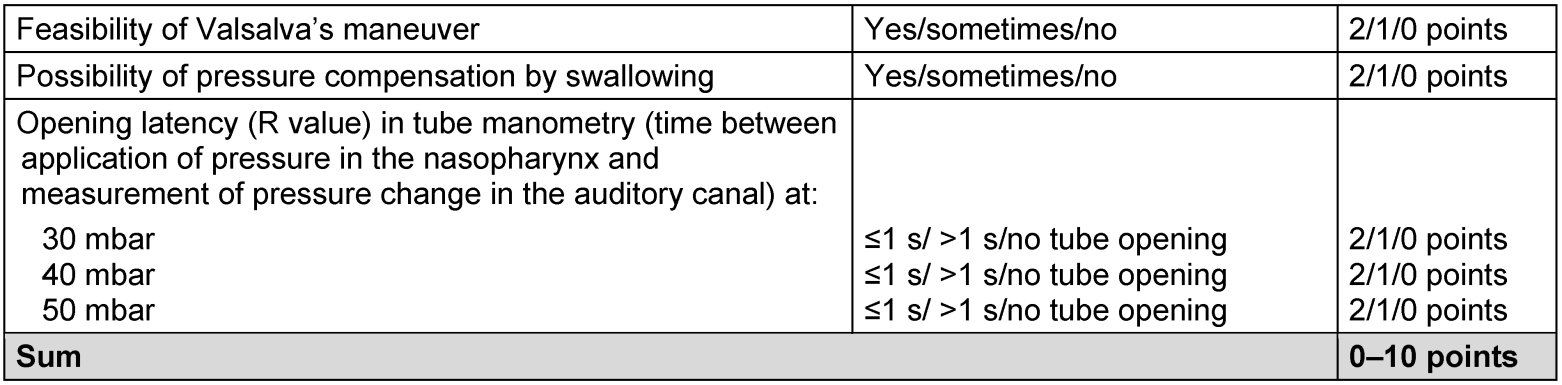
To assess the function of the Eustachian tube, a tube score was developed. It consists of feasibility of Valsalva’s maneuver, the possibility of perform pressure compensation by swallowing and the R value measured in tube manometry at 30, 40, and 50 mbar [110].

**Table 4:**
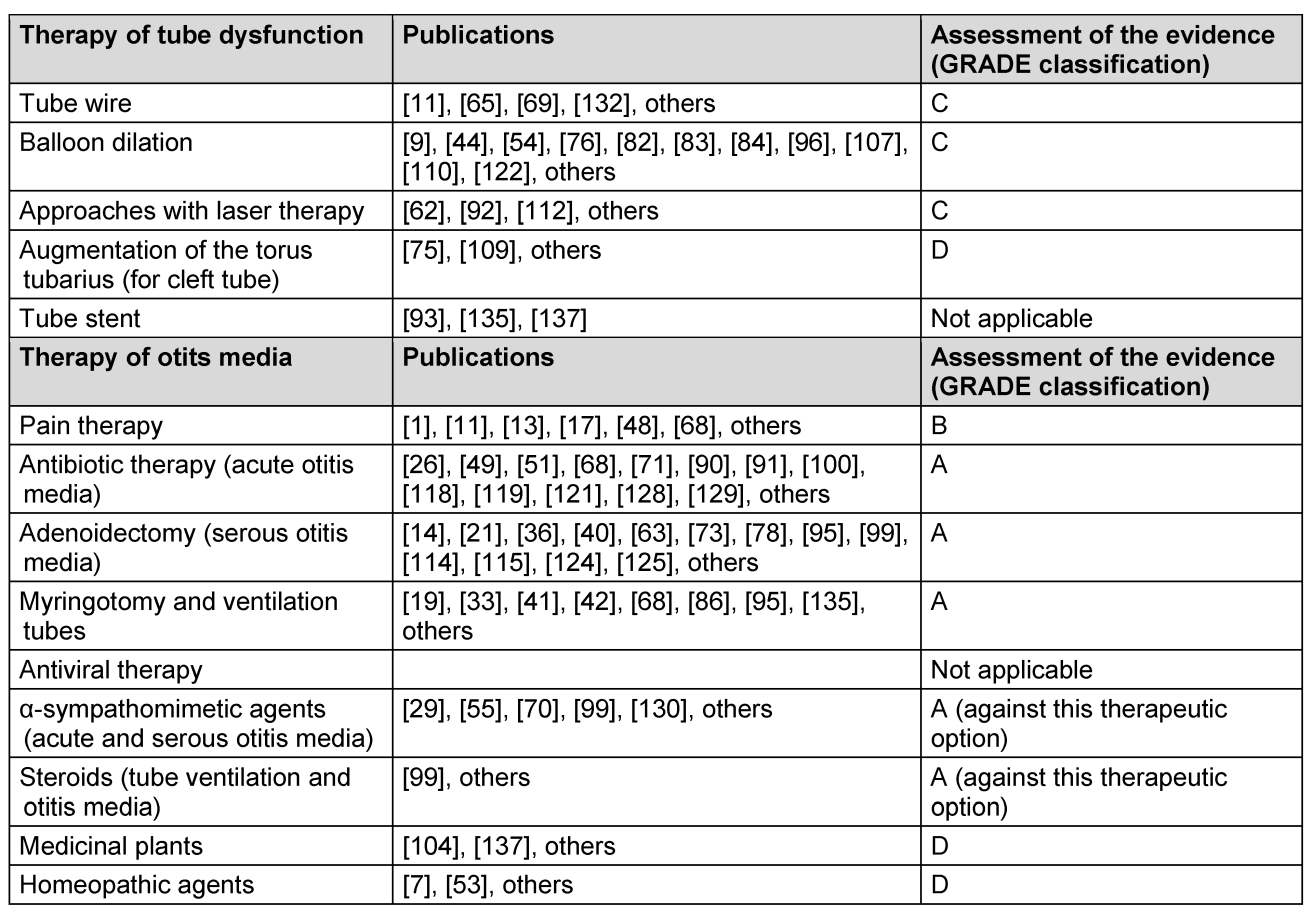
Evidence of different therapeutic approaches of disturbed middle ear ventilation and otitis media according to the GRADE classification

**Table 5:**
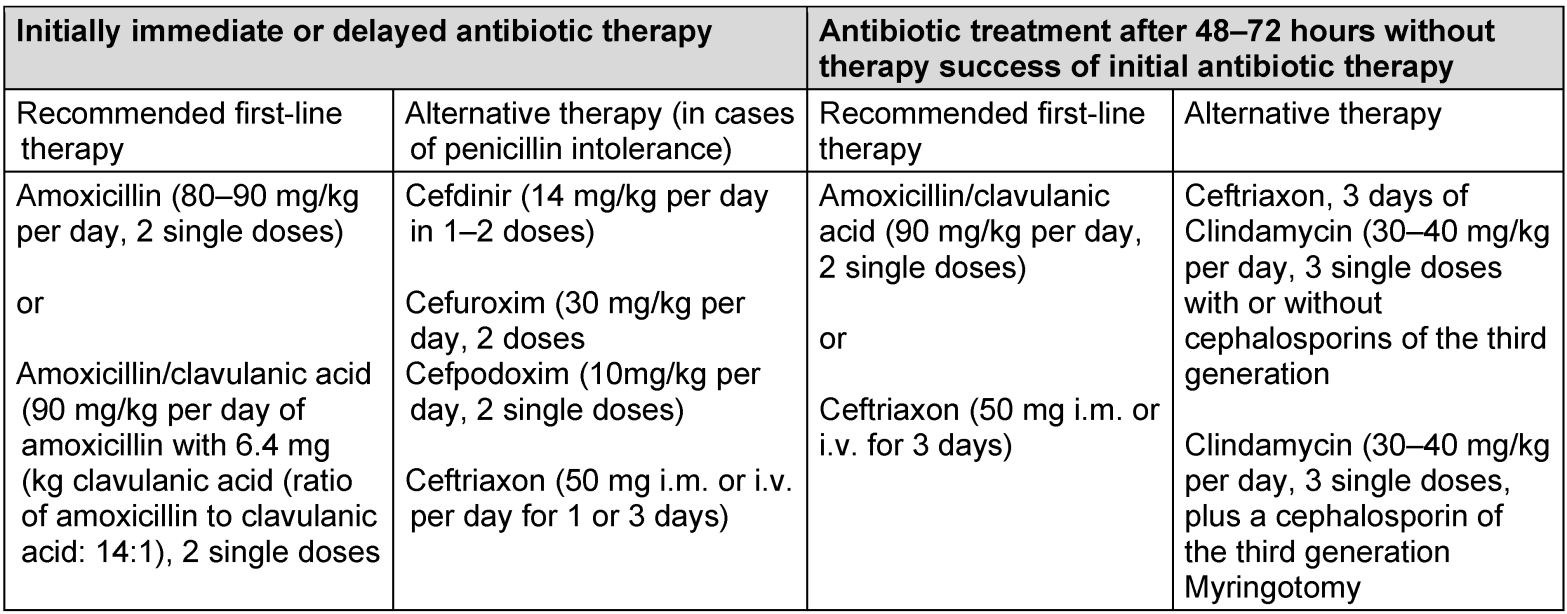
Recommended antibiotic therapy of acute otitis media according to the American Academy of Pediatrics [68]
